# Clinical and Genetic Characterization of 51 Patients with Congenital Fibrinogen Disorders from China

**DOI:** 10.1055/a-2514-7520

**Published:** 2025-01-31

**Authors:** Yaohua Cai, Hui Lu, Wenyi Lin, Yunqing Xia, Tingting Wu, Zhipeng Cheng, Liang V. Tang, Yu Hu

**Affiliations:** 1 Institute of Hematology, Union Hospital, Tongji Medical College, Huazhong University of Science and Technology, Wuhan, Hubei, China; 2 Key Lab of Molecular Biological Targeted Therapies of the Ministry of Education, Union Hospital, Tongji Medical College, Huazhong University of Science and Technology, Wuhan, Hubei, China; 3 Hubei Clinical and Research Center of Thrombosis and Hemostasis, Wuhan, Hubei, China

**Keywords:** bleeding, inherited coagulation disorders, thrombosis, gene mutations

## Abstract

**Objective:**

To investigate the classification, clinical manifestations, laboratory findings, and genetic mutations associated with hereditary fibrinogen disorders in Chinese population.

**Methods:**

Between February 2015 and February 2022, 65 patients with congenital fibrinogen disorders (CFD) were identified at Wuhan Union Hospital. Comprehensive data were available for 51 patients, allowing for a retrospective analysis.

**Results:**

The cohort comprised 17 males (33.3%) and 34 females (66.7%), with a median diagnosis age of 35.0 years (interquartile range: 25.5–42.5). Of the patients, 35 (68.6%) were diagnosed with dysfibrinogenemia, 8 (15.7%) with hypofibrinogenemia, 7 (13.7%) with hypodysfibrinogenemia, and 1 (2.0%) with afibrinogenemia. The median diagnosis ages for the asymptomatic, Grade 1, Grade 2, and Grade 3 groups were 44.5 years (range: 37–58.5), 28 years (22.5–36.5), 35.5 years (21.75–41), and 28 years (22.75–30.75), respectively. The asymptomatic group had the latest diagnosis age, whereas Grade 3 had the earliest. A negative correlation was observed between Fg:C levels and bleeding severity (rs = − 0.2937, *p* = 0.0365). In total, 52 variants were found in 51 unrelated patients, with one patient carrying two mutations. The 37 distinct mutations included 11 in FGA, 3 in FGB, and 23 in FGG.

**Conclusion:**

This study investigates the clinical, laboratory, and genetic characteristics of patients with CFD in China, revealing a negative correlation between Fg:C levels and bleeding severity. Female patients are at higher risk for gynecological complications due to physiological traits. Additionally, R35 in FGA and R301 in FGG were identified as hotspot mutations.

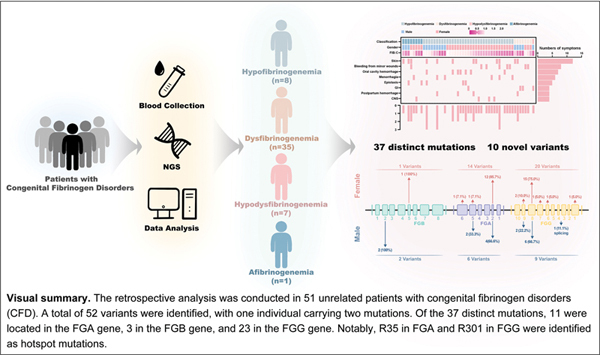

## Introduction

Hereditary fibrinogen deficiency (CFD) is a genetic disorder caused by defects in the fibrinogen genes, leading to abnormal levels and/or function of fibrinogen (Fg). The total number of amino acids of Fg is 2964. Fg is synthesized from three distinct genes—FGA, FGB, and FGG, which generate the three polypeptide chains Aa, Bβ, and γ. Fg is composed of two Aa, two Bβ, and two γ chains. Three peptide chains form elongated structures, resulting in two elongated peptide chains linked by disulfide bonds to form a symmetrical dimer. 1 2 The sequence of the three genes is FGB, FGA, and FGG, where FGB is transcribed in a direction opposite to that of FGA and FGG. FGA consists of six exons, producing various transcripts due to alternative splicing at its 3′ end. The primary transcript is composed of 610 amino acid residues encoded by exons 1 to 5 of the Aα chain; in addition, a minor transcript, the αE transcript, extends at the C-terminus via the sixth exon, encoding an additional 236 amino acid residues, ultimately forming a protein of 846 amino acids. FGB comprises eight exons, with the Bβ chain composed of 461 amino acid residues. FGG contains 10 exons, allowing for two isoforms of the γ chain to emerge due to alternative splicing; the main isoform consists of 411 amino acid residues, whereas another isoform, γ' or γB chain, consists of 427 amino acid residues.

Fibrinogen functions as coagulation factor I in the hemostatic process, converting soluble fibrinogen into insoluble fibrin through the common pathway of coagulation. 3 Fibrinogen has several binding sites for different ligands, including the binding site for tissue-type plasminogen activator (t-PA), plasminogen, and platelet glycoprotein Gp IIb/IIIa. 2 Different types of mutations and their locations lead to distinct mechanisms underlying CFDs. Mutations in the fibrinogen gene that affect specific protein domains can disrupt coagulation or fibrinolytic pathways. 4 Therefore, fibrinogen abnormalities are closely associated with thrombotic and hemorrhagic diseases. 5

The clinical manifestations of hereditary fibrinogen deficiency exhibit heterogeneity. Even with the same genetic mutation, the clinical presentations may differ. 6 Gene–gene interactions and gene–environment interactions can either exacerbate or mitigate the severity of the disease process. 7 Symptoms may range from asymptomatic to bleeding or thrombosis, with severe cases potentially leading to fatal hemorrhages, such as intracranial bleeding. Approximately 55% of patients are asymptomatic, 25% present with bleeding symptoms, and 20% experience thrombosis. Among these, about 27% of patients have both bleeding and thrombotic symptoms. 8 Bleeding symptoms may worsen in women of childbearing age and postpartum. 9 The frequency of miscarriage is relatively high among female patients, highlighting the crucial role of fibrinogen in maintaining hemostasis throughout pregnancy. 10

This study presents a comprehensive analysis of 51 cases of CFD, focusing on various aspects such as genotypes, laboratory phenotypes, and clinical symptoms. Additionally, we utilized bioinformatics tools to explore the disease potential associated with the genetic variants. This approach allowed us to investigate how these mutations correlate with the clinical features, providing deeper insights into the pathophysiology of CFD and its implications for patient management and treatment strategies.

## Methods

### Data Collection

Between February 2015 and February 2022, 65 patients with laboratory phenotypes suggestive of CFD were initially diagnosed at Wuhan Union Hospital. After collecting clinical information and confirming the diagnosis through genetic testing, 51 unrelated patients with complete data were included in the study. The inclusion criteria were based on the laboratory diagnostic procedures for CFD as established by the International Society on Thrombosis and Haemostasis. 11 The study was approved by the Ethics Committee of Wuhan Union Hospital. All participants provided informed consent after receiving comprehensive information, in accordance with the Declaration of Helsinki.

Clinical data were recorded, including the patient's bleeding history and venous or arterial thrombosis history. The European network of rare bleeding disorders bleeding score system (EN-RBD-BSS) were employed to assess the patient's bleeding severity. 12

### Coagulation Tests

Peripheral venous blood samples were collected and mixed with 0.109 mol/L sodium citrate in a 1:9 ratio for anticoagulation. The samples were then centrifuged at 3700 rpm for 10 minutes. The resulting platelet-poor plasma was used for coagulation assays, which were completed within 2 hours. Plasma coagulation parameters were collected, including activated partial thromboplastin time (aPTT; normal range: 25–37 seconds), prothrombin time (PT; normal range: 11–14 seconds), thrombin time (TT; normal range: 12–16 seconds), fibrinogen activity (Fg:C; normal range: 1.8–3.5 g/L), and fibrinogen antigen (Fg:Ag; normal range: 2.1–4 g/L). Fg:C was measured using the Clauss method with the STA-R automated coagulometer (Stago, France), whereas Fg:Ag levels were determined using the enzyme-linked immunosorbent assay method. Blood cells were stored at −80°C. 13 Genomic DNA was extracted from peripheral blood cells using the QIAamp DNA Blood Mini Kit (250) for genomic DNA extraction. The laboratory findings for hypofibrinogenemia are characterized by Fg:C/Fg:Ag greater than 0.7. The diagnostic criteria for dysfibrinogenemia are Fg:C/Fg:Ag below 0.7 with normal FIB:Ag. Hypodysfibrinogenemia is characterized by Fg:C/Fg:Ag below 0.7 with decreased Fg:Ag. The diagnostic criteria for afibrinogenemia are extremely low or undetectable fibrinogen (<0.1 g/L). 14 15

### Genetic Analysis

A custom-designed next-generation sequencing (NGS) panel was utilized for genetic testing. Targeted NGS was performed to analyze the protein-coding regions along with 10 base pairs of adjacent intronic sequences, using the Illumina platform for sequencing. Polymerase chain reaction and Sanger sequencing were conducted on DNA from selected patients to validate the genetic variants identified by NGS. All mutations described in this study are annotated following the Human Genome Variation Society nomenclature, based on the reference transcripts for each gene (FGA:NM_000508, FGB:NM_005141, and FGG:NM_000509). Previously reported variants associated with CFD were identified using the Human Gene Mutation Database (HGMD; https://www.hgmd.cf.ac.uk/ac/index.php ), the Clinical Variant Database (ClinVar; https://www.ncbi.nlm.nih.gov/clinvar/ ), and the European Association for Haemophilia and Allied Disorders-Coagulation Factor Variant Databases (EAHAD-CFDB; https://dbs.eahad.org/ ). Pathogenicity of the novel variants was assessed using multiple prediction tools, including SIFT, PolyPhen, Mutation Assessor, MetaLR, MutationTaster, MutationAssessor, and CADD. Potential splicing mutations were further evaluated using SpliceAI.

### Statistical Analysis

Quantitative data were expressed as either the median (interquartile range, IQR) or the mean ± standard deviation, whereas qualitative data were reported as counts and percentages. The EN-RBD bleeding scores were assigned as follows: 0 for asymptomatic, 1 for Grade I, 2 for Grade II, and 3 for Grade III bleeding. The Shapiro–Wilk test was used to assess the normality of the data distribution. For non-normally distributed data, Spearman's rank correlation coefficient was calculated to assess the strength and direction of the association between the EN-RBD score and Fg:C. The Kruskal–Wallis H test was applied to analyze differences between groups, followed by pairwise comparisons with Dunn's correction for multiple testing. Fisher's exact test was employed to compare categorical variables across groups, with a *p* -value of less than 0.05 was considered statistically significant. Ordinal logistic regression was performed to assess the impact of various variables on bleeding severity. All statistical analyses and visualizations were performed using R Studio version 4.2.1, SPSS version 29.0.1.0 and GraphPad Prism 10.

## Results

### Demographics and Clinical Characteristics

A total of 65 patients diagnosed with congenital fibrinogen disorders (CFDs) were identified at Wuhan Union Hospital between February 2015 and February 2022. Of these, detailed clinical information and genetic results were available for 51 patients, enabling a retrospective analysis. The cohort consisted of 17 males (33.3%) and 34 females (66.7%). Demographic and laboratory data are presented in Table 1 . Based on Fg:C and antigen levels Fg:Ag, the patients were classified into four categories: Dysfibrinogenemia ( *n* = 35, 68.6%), Hypofibrinogenemia ( *n* = 8, 15.7%), Hypodysfibrinogenemia ( *n* = 7, 13.7%), and Afibrinogenemia ( *n* = 1, 2.0%). The overall median age at diagnosis was 35.0 years (IQR: 25.5–42.5). The median age in the Hypofibrinogenemia group was 26.5 years (IQR: 21.5–36.5), in the Dysfibrinogenemia group was 39 years (IQR: 29–45), and in the Hypodysfibrinogenemia group was 23 years (IQR: 22.5–37.5). The Kruskal–Wallis analysis revealed no statistically significant differences in age across the three groups.

**Table 1:** Demographic and laboratory data

	Hypofibrinogenemia ( *n* = 8)	Dysfibrinogenemia ( *n* = 35)	Hypodysfibrinogenemia ( *n* = 7)	Afibrinogenemia ( *n* = 1)	All cases ( *n* = 51)
Female, *N* (%)	5 (62.5%)	26 (74.3%)	3 (42.9%)	0 (0.0%)	34 (66.7%)
Age at diagnosis (y), median (IQR)	26.5 (21.5–36.5)	39 (29–45)	23 (22.5–37.5)	28	35.0 (25.5–42.5)
Age at inclusion (y), median (IQR)	28 (21.5–40)	39 (33–44.75)	23 (22.5-37.5)	28	36.5 (28–42.75)
Bleeding severity, *n* (%)					
Asymptomatic	2 (25.0%)	11 (31.4%)	1 (14.3%)	–	14 (27.5%)
Grade 1	2 (25.0%)	9 (25.7%)	–	–	11 (21.6%)
Grade 2	2 (25.0%)	12 (34.3%)	3 (42.9%)	–	17 (33.3%)
Grade 3	2 (25.0%)	3 (8.6%)	3 (42.9%)	1 (100%)	9 (17.6%)
Thrombosis, *n* (%)	1 (12.50%)	4 (11.43%) a	2 (28.57%) a	1 (100%) a	8 (15.69%)
Laboratory characteristics (mean ± SD)					
Fg:C (g/L)	0.64 ± 0.26	0.82 ± 0.36	0.73 ± 0.30	0.4	0.77 ± 0.34
Fg:Ag (g/L)	1.04 ± 0.456	2.78 ± 0.74	0.96 ± 0.33	0.56	2.20 ± 1.02
aPTT (s)	37.48 ± 5.32	33.81 ± 5.87	37.93 ± 2.53	38.1	35.04 ± 5.68
PT (s)	15.16 ± 1.62	13.44 ± 1.58	14.52 ± 1.39	22.8	14.05 ± 2.10
TT (s)	28.72 ± 9.01	22.58 ± 5.62	23.47 ± 4.36	21.3	23.65 ± 6.51

Abbreviations: aPTT, activated partial thromboplastin time; Fg:C, fibrinogen activity; Fg:Ag, fibrinogen antigen; PT, prothrombin time; TT, thrombin time.

a One patient presented with both thrombotic and bleeding symptoms.

**Fig. 1:**
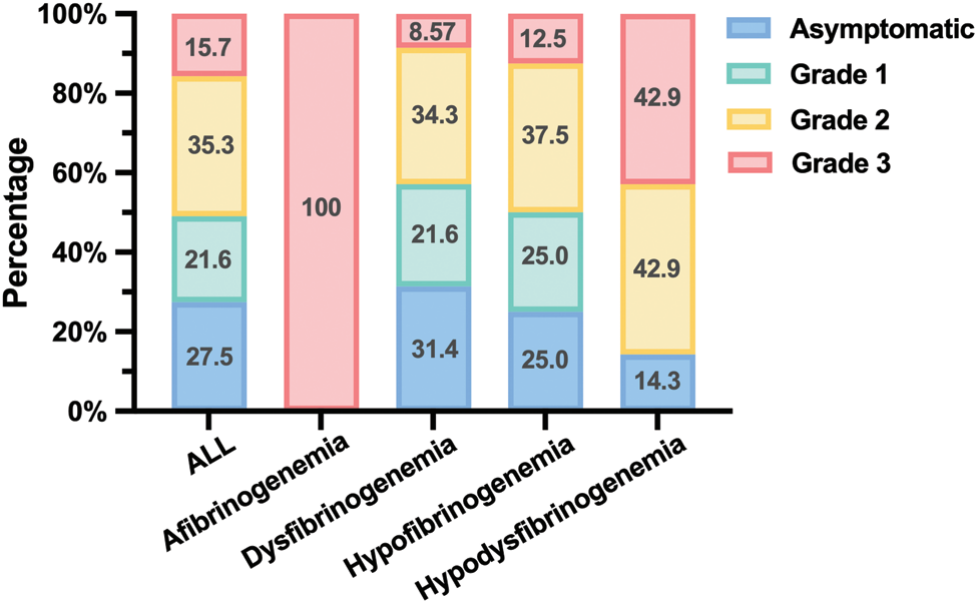
Proportion of bleeding severity in different groups. Proportion of bleeding severity in different groups. Categorization of 51 patients by disease type and description of proportion of bleeding severity, the first column represents the results for all patients.

Bleeding symptoms in patients were categorized using the EN-RBD classification. 12 Fig. 1 illustrates the distribution of bleeding severity across different diagnostic groups, Among the patients, 14 (27.5%) were asymptomatic (no documented bleeding episodes), 11 (21.6%) experienced Grade 1 bleeding, 17 (33.3%) had Grade 2 bleeding, and 8 (15.6%) presented with Grade 3 bleeding. The proportion of patients with more severe bleeding progressively increased across the groups of Dysfibrinogenemia, Hypofibrinogenemia, and Hypodysfibrinogenemia. The median age at diagnosis for the four groups, categorized as Asymptomatic, Grade 1, Grade 2, and Grade 3 bleeding, was 44.5 years (37–58.5), 28 years (22.5–36.5), 35.5 years (21.75–41), and 28 years (22.75–30.75), respectively. The Asymptomatic group had the latest median diagnosis age, whereas the Grade 3 group had the earliest. Statistically significant differences in the median age were observed between the Asymptomatic, Grade 1, and Grade 3 groups ( *p* = 0.0129 and 0.0074, respectively; Supplementary Fig. S1 , available in the online version).

**Fig. 2:**
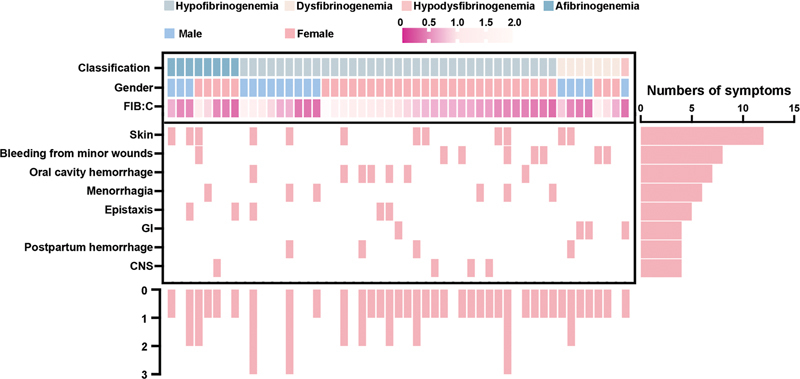
Oncoplot displays clinical information and bleeding symptoms of patients. The upper half of the middle panel shows the disease classification, gender, and Fg:C of the patients. The lower half of the middle panel presents specific bleeding manifestations of the patients. The bottom panel displays the total number of bleeding symptoms for each patient. The right panel illustrates the distribution of different bleeding symptoms.

Clinical information from 51 patients was collected, and 10 cases (19.6%) had a family history. Of the 14 patients without bleeding symptoms, 6 cases (11.7%) were identified through preoperative examinations, 4 cases (7.8%) were found during medical consultations for other conditions, and 4 cases (7.8%) were diagnosed due to thrombotic symptoms. Among the 35 patients (72.5%) with bleeding symptoms, 10 experienced bleeding in multiple locations (≥2), with skin bleeding being the most frequent symptom, observed in 12 patients (34.2%). The next most common symptoms were menorrhagia ( *n* = 8, 22.9%), bleeding from minor wounds ( *n* = 7, 20.0%), oral cavity hemorrhage ( *n* = 6, 17.1%), and epistaxis ( *n* = 5, 14.3%). Four patients experienced gastrointestinal bleeding (including 2 cases of hemorrhoidal bleeding), postpartum hemorrhage, and intracranial hemorrhage, respectively ( Fig. 2 ).

### Gender Differences in Clinical Characteristics

**Fig. 3:**
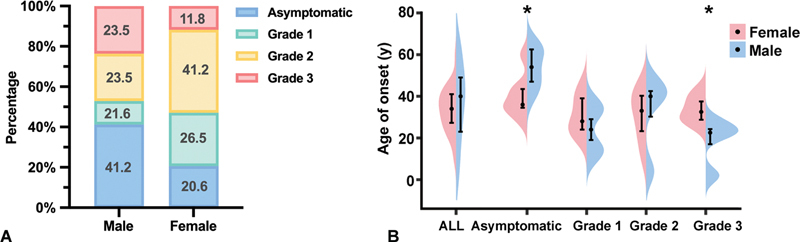
Gender-based analysis of bleeding severity and age at diagnosis. ( **A** ). Proportion of bleeding severity in different gender. ( **B** ). Age at diagnosis in different grades. Statistical significance was considered at a *p* -value < 0.05. The symbol “*” indicates *p* -values < 0.05.

In 34 female patients, the proportion of Grade 2 bleeding was the highest ( *n* = 14, 41.2%), as most women presented with excessive menstrual bleeding and were classified as Grade 2 ( *n* = 8, 22.9%; Fig. 3A ). With regard to pregnancy and complications, 5 patients had spontaneous miscarriages, and postpartum hemorrhage was observed in 4 cases, including 3 vaginal deliveries and 1 cesarean section. A higher proportion of males (41.2%) were asymptomatic for bleeding, and 4 patients sought medical attention for venous thromboembolism. Additionally, three other patients were diagnosed with coagulation abnormalities during preoperative examinations. Overall, the age of diagnosis showed no significant difference between males and females, although asymptomatic males ( *n* = 7) were diagnosed later than their female counterparts ( *n* = 7; Fig. 3B ).

### Laboratory Characteristics

**Fig. 4:**
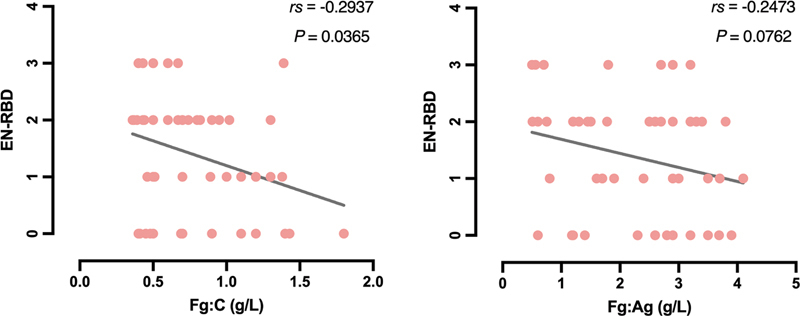
Correlation analysis of Fg:C and Fg:Ag with EN-RBD-BSS Score. Correlation analysis of Fg:C and Fg:Ag with the EN-RBD-BSS Score. EN-RBD-BSS, the European network of rare bleeding disorders bleeding score system.

In the 51 patients, the main laboratory findings were a marked reduction in Fg:C and an extended TT. The initial coagulation profile of the 51 patients showed the following median values: Fg:C 0.77 ± 0.34 g/L, Fg:A 2.20 ± 1.02 g/L, aPTT 35.04 ± 5.68 s, TT 23.65 ± 6.51 s, PT 14.05 ± 2.10 s. Spearman's rank correlation was employed to evaluate the association between Fg:C levels and bleeding grades. The analysis revealed a negative correlation between Fg:C levels and the bleeding severity score (rs = − 0.2937, *p* = 0.0365; Fig. 4A ). While, the relationship between Fg:Ag levels and the bleeding severity score was not significant (rs = − 0.2473, *p* = 0.0762; Fig. 4B ).

### Spectrum of Mutation

**Fig. 5:**
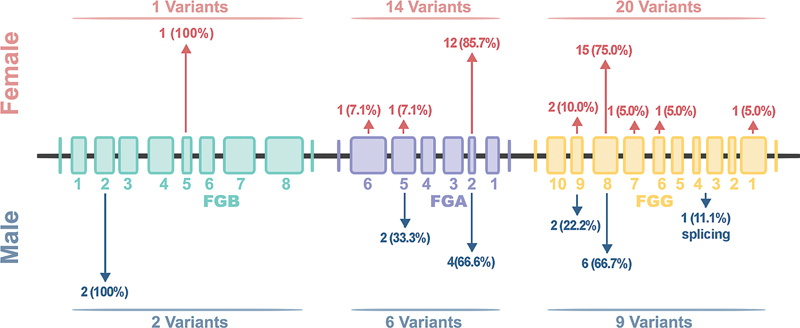
Mutation distribution map. The upper section displays mutations detected in females, whereas the lower section shows those detected in males. The height of the arrows indicates the frequency of mutation occurrence.

**Fig. 6:**
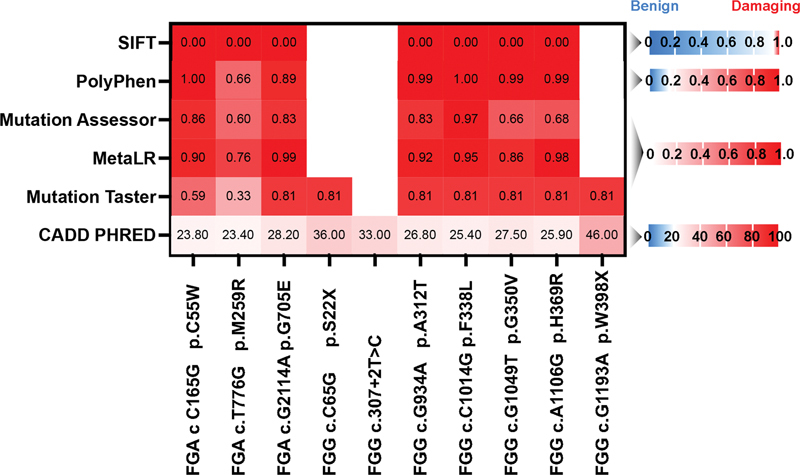
In silico prediction of novel variants. The biological impact of 10 new mutations was assessed using in silico tools. These tools included Sorting Intolerant from Tolerant (SIFT), Polymorphism Phenotyping (PolyPhen), Mutation Assessor rankscore, Meta-Learning for Predicting the Likelihood of Pathogenicity of Variants (MetaLR) rankscore, Mutation Taster converted rankscore, Combined Annotation Dependent Depletion (CADD) PHRED. The deleterious threshold is determined based on the literature. 43

A total of 52 gene mutations were detected among 51 unrelated patients ( Fig. 5 ). One individual carried two different mutations. A total of 37 distinct gene mutations were observed (11 in the FGA gene, 3 in the FGB gene, and 23 in the FGG gene; refer to Table 2 for details). This comprised 1 homozygous frameshift insertion, 1 splicing mutation, 31 heterozygous missense mutations, and 3 stop codon mutations. The patient with afibrinogenemia exhibited a homozygous mutation in FGA: c.1483dup.M495Nfs*18. The FGG gene exhibited the most frequent mutations, occurring in 29 cases (56.9%), followed by FGA and FGB, with mutations found in 19 cases (37.3%) and 3 cases (5.9%), respectively. Exon 8 of FGG and exon 2 of FGA were noted as locations with a high frequency of mutations. Of the mutations, 21 (40.38%) were located in exon 8 of the FGG gene, and 16 (30.77%) were present in exon 2 of the FGA gene. The common hotspot mutations identified included: FGA:exon2:c.G104A:p.R35H ( *n* = 8) and FGG:exon8:c.C901T:p.R301C ( *n* = 5). We discovered a total of 10 novel variants. Fig. 6 illustrates the potential pathogenic effects of new variants assessed through different in silico tools. Most tools predict the mutations to be harmful. FGG c.307 + 2T > C represents a splicing mutation, predicted by SpliceAI to suggest Splice Loss, with a splice Δ score of 0.99. This indicates a strong possibility that the variant has a significant effect on splicing. This T > C substitution at the donor site has the potential to interfere with the spliceosome's recognition of the splice site, leading to donor loss.

**Table 2:** Genotype identified in patients

Gene	cDNA	Protein mutation	Exon	Intron	Type of mutation	Genotype	Numbers of patients	Asymptomatic/Bleeding	Novelty
FGA	c.G92T	p.G31V	2		Missense	Hetero	1	0/1	–
	c.C103A	p.R35S	2		Missense	Hetero	2	0/2	–
	c.C103G	p.R35G	2		Missense	Hetero	1	0/1	–
	c.G104A	p.R35H	2		Missense	Hetero	8	1/7	–
	c.A112G	p.R38G	2		Missense	Hetero	2	0/2	–
	c.T163G	p.C55G	2		Missense	Hetero	1	0/1	–
	c.C165G	p.C55W	2		Missense	Hetero	1	0/1	√
	c.T776G	p.M259R	5		Missense	Hetero	1	0/1	√
	c.G1002A	p.W334X	5		Nonsense	Hetero	1	1/0	–
	c.1483dup	p.M495Nfs*18	5		Frameshift insertion	Homo	1	0/1	–
	c.G2114A	p.G705E	6		Missense	Hetero	1	0/1	√
FGB	c.C130T	p.R44C	2		Missense	Hetero	1	1/0	–
	c.G292A	p.A98T	2		Missense	Hetero	1	1/0	–
	c.C617T	p.P206L	5		Missense	Hetero	1	0/1	–
FGG	c.C65G	p.S22X	1		Nonsense	Hetero	1	0/1	√
	c.307 + 2T > C			2	Splice site	Hetero	1	0/1	√
	c.G571A	p.G191R	6		Missense	Hetero	1	0/1	–
	c.A766T	p.N256Y	7		Missense	Hetero	1	1/0	–
	c.A863C	p.Y288S	8		Missense	Hetero	1	0/1	–
	c.C901T	p.R301C	8		Missense	Hetero	5	0/5	–
	c.G902A	p.R301H	8		Missense	Hetero	2	1/1	–
	c.G934A	p.A312T	8		Missense	Hetero	1	0/1	√
	c.C944G	p.A315G	8		Missense	Hetero	1	1/0	–
	c.C944T	p.A315V	8		Missense	Hetero	1	1/0	–
	c.G952A	p.G318S	8		Missense	Hetero	1	1/0	–
	c.C1014G	p.F338L	8		Missense	Hetero	1	0/1	√
	c.C1032A	p.D344E	8		Missense	Hetero	1	1/0	–
	c.A1033T	p.N345Y	8		Missense	Hetero	1	1/0	–
	c.G1049T	p.G350V	8		Missense	Hetero	1	1/0	√
	c.G1099A	p.A367T	8		Missense	Hetero	2	1/1	–
	c.G1099C	p.A367P	8		Missense	Hetero	1	1/0	–
	c.A1106G	p.H369R	8		Missense	Hetero	1	0/1	√
	c.A1112G	p.N371S	8		Missense	Hetero	1	0/1	–
	c.T1173A	p.N391K	9		Missense	Hetero	1	0/1	–
	c.G1174C	p.G392R	9		Missense	Hetero	1	0/1	√
	c.G1193A	p.W398X	9		Nonsense	Hetero	1	0/1	√
	c.C1201T	p.R401W	9		Missense	Hetero	1	0/1	–

Abbreviations: Hetero, heterozygous; Homo, homozygous.

### Thrombosis Events in Congenital Fibrinogen Disorder

In this study, we identified eight patients with CFDs who developed thrombotic complications ( Table 3 ). Pulmonary embolism (PE) was observed in two cases: a patient with hypofibrinogenemia (P1) and another with dysfibrinogenemia (P2). Deep vein thrombosis (DVT) was detected in two patients: one with dysfibrinogenemia (P4) presenting with right leg DVT and left frontal lobe infarction, and another with dysfibrinogenemia (P5), who experienced left leg DVT accompanied by PE. Cerebral venous sinus thrombosis (CVST) was documented in four cases, including patients with hypodysfibrinogenemia (P6, P7), afibrinogenemia (P8), and dysfibrinogenemia (P3). Three of these patients experienced concurrent intracranial hemorrhages: left temporal lobe hemorrhage (P6), left frontoparietal hemorrhage (P7), and left temporoparietoinsular hemorrhage with periventricular bleeding and subfalcine herniation (P3).

**Table 3:** Case reports of patients with thrombosis

Patient ID	Age at diagnosis (y)	Sex	Diagnosis	Bleeding symptoms	Thrombotic events	CFD associated mutation	Other mutations
P1	57	M	Hypoﬁbrinogenemia	–	PE	FGA:c.G1002A: p.W334X	PROS1:c.A149C:p.K50T
P2	35	M	Dysfibrinogenemia	–	PE	FGB:c.C130T:p.R44C	
P3	42	F	Dysfibrinogenemia	Left temporo-parieto-insular lobe hemorrhage with periventricular bleeding, accompanied by subfalcine herniation	Thrombosis in the left transverse sinus and sigmoid sinus	FGB:c.C617T:p.P206L	
P4	63	M	Dysfibrinogenemia	–	DVT in right leg, left frontal lobe cerebral infarction	FGB:c.G292A:p.A98T	PROS1:c.-168C > T
P5	40	M	Dysfibrinogenemia	–	DVT in left leg, PE	FGG:c.G1099A:p.A367T	PROC:c.G1218A:p.M406I, PROS1:c.G947A:p.R316H
P6	22	M	Hypodysﬁbrinogenemia	Left temporal lobe hemorrhage	Left sigmoid sinus thrombosis	FGA:c.A112G:p.R38G	
P7	23	M	Hypodysﬁbrinogenemia	Left frontoparietal lobe hemorrhage	Thrombosis in the left transverse-sigmoid sinus	FGG:c.307 + 2T > C	
P8	28	M	Aﬁbrinogenemia	Left frontoparietal lobe hemorrhage	Thrombosis in the left superior cerebral vein and superior sagittal sinus	FGA:c.1483dup:p.M495Nfs*18	

Abbreviations: DVT, deep vein thrombosis; F, female; M, male; PE, pulmonary embolism.

**Fig. 7:**
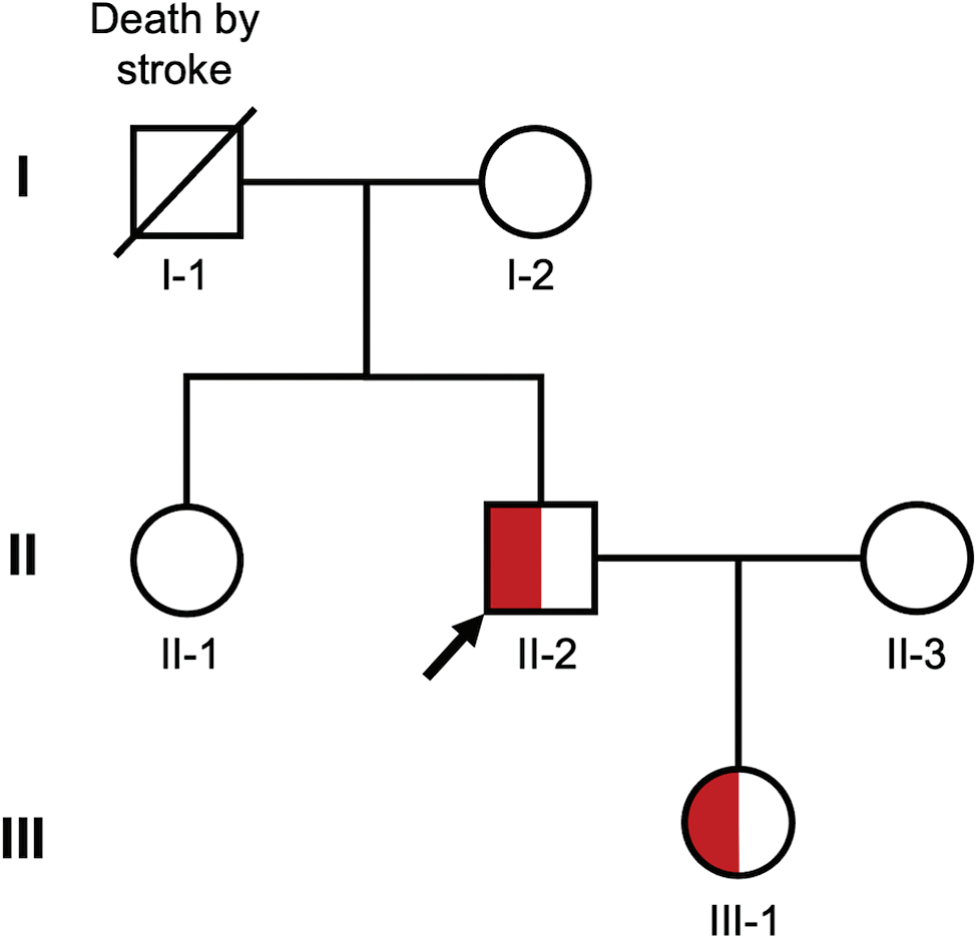
Pedigree of the family with FGB.C130T:p.R44C variant. This pedigree chart illustrates the family structure and inheritance pattern of the bleeding disorder. Red-filled symbols indicate individuals carrying the FGB.C130T:p.R44C variant. The arrow points to the proband, the individual from whom the study originated.

A family study was conducted on the proband (II-2), who carries the FGB.C130T:p.R44C variant, previously associated with thrombosis ( Fig. 7 ). The proband's mother (I-2) and sister (II-1) do not carry the mutation, whereas the proband's daughter (III-1) does. The proband presented with decreased fibrinogen activity (Fg:C) and prolonged TT, consistent with prior clinical observations ( Table 4 ). Interestingly, despite carrying the same mutation, the proband's daughter has not exhibited any thrombotic or bleeding events, although she experiences occasional menstrual irregularities. This family study highlights the clinical variability associated with the FGB.C130T:p.R44C variant, suggesting that other genetic-, environmental-, or sex-related factors may influence the clinical manifestation of the mutation.

**Table 4:** The coagulation results of the family study

Family member	Fg:C (g/L)	Fg:Ag (g/L)	aPTT (s)	PT (s)	TT (s)
I-2	2.5	3.2	29.8	12.6	13.4
II-1	1.8	3.7	30.9	13.5	15.8
II-2	0.9	3.5	37.8	16.4	18.7
II-3	3.1	3.7	32.8	12.2	14.1
III-1	1.3	3.4	34.2	13.2	17.2

Abbreviations: aPTT, activated partial thromboplastin time; Fg:Ag, fibrinogen antigen; Fg:C, fibrinogen activity; PT, prothrombin time; TT, thrombin time.

## Discussion

This study presents a retrospective analysis involving 51 unrelated CFD patients. It is the largest study to date in Asia regarding the number and types of mutations associated with CFD. The aim is to describe the genetic landscape of CFD associated mutation in China. The main clinical manifestation of CFDs is bleeding, as 72.5% of the participants reported having bleeding episodes. In our cohort, there are differences in the age of diagnosis among patients with varying severity of bleeding. Those with severe bleeding manifestations were diagnosed at a younger age compared with asymptomatic patients, who had a later age of diagnosis.

In CFD patients, coagulation tests usually reveal a marked extension of TT, whereas aPTT and PT are generally normal or slightly prolonged. 16 17 While the PT algorithm frequently indicates normal fibrinogen levels, the Clauss method reveals a notable decrease in fibrinogen activity. 18 19 A definitive diagnosis of CFD can be made after excluding factors that may lead to secondary Fg reduction, such as liver disease and Fg consumption disorders. 20 The measurement of fibrinogen concentration serves as an important preliminary screening tool for identifying coagulation disorders, with primary methods including physicochemical, immunological, and coagulation protein assays. The measurement of fibrinogen concentration is a crucial initial screening tool for identifying coagulation dysfunction, with primary methods including physicochemical techniques, immunological methods, and clotting protein assays. The Clauss method is the standard test for fibrinogen recommended by the National Committee for Clinical Laboratory Standards. It allows for a more accurate assessment of fibrinogen's functional activity. 20 21 Our analysis revealed a notable negative correlation between Fg:C levels and the EN-RBD score, suggesting that reduced Fg:C levels correspond to an increased risk of bleeding. While Fg:Ag levels did not show a significant correlation with the EN-RBD score. These results align with earlier studies, 12 22 highlighting the critical significance of fibrinogen functional activity relative to its antigen concentration when evaluating bleeding risk. Nevertheless, the laboratory diagnosis of CFD remains challenging. In a study involving 101 CD patients, the results of TT and RT (reptilase time) tests showed that 87.6 and 89.7% of patients experienced prolongation, respectively. 9 However, certain patients exhibited normal TT, and some even showed shortened TT. 23 These findings suggest that laboratory testing for CFD is not always reliable, potentially leading to missed diagnoses or misdiagnoses. Thus, genetic testing is essential to enhance detection rates and provide a more comprehensive diagnostic approach.

Fibrinogen disorders exhibit significant clinical and laboratory heterogeneity, complicating diagnosis and management. For instance, three asymptomatic patients in our study showed disproportionately prolonged TT (>30 seconds), highlighting the disconnect between laboratory abnormalities and clinical symptoms. Mechanisms such as compensatory pathways, mutation-specific effects on fibrin polymerization, and additional triggers like trauma may contribute to this variability. Furthermore, the same mutation (FGG:c.G1099A:p.A367T) led to contrasting outcomes in two patients: a 28-year-old female experienced severe postpartum hemorrhage, whereas a 40-year-old male presented with thrombotic complications, including DVT and PE. Additional genetic testing revealed the male patient also harbored prothrombotic mutations ( *PROC* :NM_000312:exon9:c.G1218A:p.M406I and *PROS1* :NM_000313:exon9:c.G947A:p.R316H), likely contributing to his hypercoagulable state. These contrasting outcomes highlight the intricate interplay between fibrinogen mutations, other genetic modifiers, regulatory mechanisms, and potentially sex-specific factors, reinforcing the complexity of genotype–phenotype correlations in CFD.

To date, 526 genetic mutation sites associated with CFD have been identified, with the highest number of mutations occurring in FGA (189 types) and the lowest in FGB (140 types). 24 Mutations are mainly missense mutations, with frameshift mutations, nonsense mutations, and large deletions following. Fibrinogen gene mutations can result in fibrinogen deficiency by influencing mRNA splicing or stability and by affecting various mechanisms related to protein synthesis, assembly, and release. 25 In this study, 35.3% of patients ( *n* = 15) carried hotspot variants (p.R35C/H and p.R301C/H), which is consistent with previously reported hotspot mutations. 22 26 27 Among the 15 patients, 14 exhibited bleeding symptoms, with only one being asymptomatic, and none experienced thrombotic events. Previous research has shown variability in the clinical presentations associated with these two hotspot mutations. 9 26 28 Consequently, there is no definitive evidence linking these hotspot mutations to thrombotic phenotypes.

The same mutation, FGG:NM_000509:exon8:c.G1099A:p.A367T, was associated with contrasting clinical phenotypes in two patients. A 28-year-old female experienced severe postpartum hemorrhage, whereas a 40-year-old male was admitted for thrombotic complications (DVT and PE). Further screening revealed that the male patient carried additional prothrombotic mutations (PROC:NM_000312:exon9:c.G1218A:p.M406I and PROS1:NM_000313:exon9:c.G947A:p.R316H), likely contributing to his hypercoagulable state. These contrasting outcomes may result from the interplay of fibrinogen mutations with other genetic modifiers, regulatory mechanisms, or sex-specific factors, highlighting the complexity of genotype–phenotype correlations.

In some patients with CFD, thrombosis can occur despite prolonged clotting times and reduced fibrinogen levels. Some fibrinogen variants markedly elevate the risk of thrombus formation, resulting in carriers having a strong positive family history and occurrences of thrombotic events, including thrombosis in peripheral arteries, cerebral veins, and hepatic veins. 16 29 30 Several mechanisms may contribute to this paradox. First, certain CFD-related mutations produce structurally abnormal fibrinogen molecules, which, despite their impaired function, can form dense, degradation-resistant fibrin networks that increase thrombosis risk. 9 Second, impaired fibrinolysis plays a key role, involving resistance to plasmin-mediated lysis or defective binding of t-PA. 31 For example, fibrinogen New York I (Bβ-chain 9–72 amino acid deletion) impairs fibrin–thrombin binding, reducing plasminogen activation and leading to fibrinolytic dysfunction. 32 Similarly, fibrinogen Dusart (Paris V) and Chapel Hill III mutations (Aα-chain Arg554Cys) hinder fibrin–plasminogen interactions, limiting t-PA-mediated activation of plasminogen and further compromising fibrinolysis. 33 34 Finally, elevated circulating thrombin levels may exacerbate thrombosis. Under normal conditions, thrombin facilitates fibrin formation while binding antithrombin to prevent excessive clotting. However, certain mutations, such as fibrinogen Naples (Bβ-chain Ala68Thr), block fibrin–thrombin binding, increasing free thrombin levels in circulation and promoting thrombosis. 35 These mechanisms underscore the complexity and heterogeneity of thrombosis in CFD patients.

This report describes eight patients who were hospitalized due to thrombotic events. We conducted genetic screening to exclude other potential genetic mutations that could cause thrombosis. The results showed that three patients had combined PROC or PROS1 mutations, whereas the remaining five did not carry any thrombosis-associated genetic mutations. A 22-year-old male patient with fibrinogen deficiency had a homozygous FGA: p.M495Nfs*18 mutation, presenting with bleeding symptoms and experiencing a hemorrhagic stroke. This patient was hospitalized with complaints of “sudden dizziness for 3 days and worsening right-sided weakness for 2 days” and examinations indicated acute infarction in the left hemisphere along with coagulation dysfunction. Additionally, three patients with hypofibrinogenemia were diagnosed with CVST accompanied by bleeding, with genotypes as follows FGG:c.307 + 2T > C,FGA:c.A112G:p.R38G, FGB:c.C617T:p.P206L. Moreover, a patient admitted for PE carried the FGB:c.C130T:p.R44C mutation. Numerous studies have documented the link between this mutation site and thrombotic events. For instance, one study mentioned a 37-year-old female who experienced thrombotic strokes at ages 29 and 37. 19 Another study reported a case of a female who suffered from a PE postpartum. 9 Two subjects carrying the FGB R44C mutation experienced DVT. 28 The research found this mutation site in patients with thrombosis and isolated fibrinogen from their samples. Findings showed that the mutated cysteine residue created a disulfide bond complex with albumin, which then circulated in the blood, increasing the thrombotic risk. 36

We additionally analyzed the impact of coagulation-related gene mutations and blood type on bleeding severity ( Supplementary Fig. S2 , available in the online version). The ordinal logistic regression analysis revealed that the F12 mutation was significantly associated with increased bleeding severity (odds ratio [OR] = 6.62, *p* = 0.03), suggesting a strong effect on bleeding risk. In contrast, the SERPINC1 mutation was linked to a reduced likelihood of severe bleeding (OR = 0.18), likely due to its role in promoting thrombosis. Although no significant associations were found between ABO blood groups and bleeding severity, group O showed a trend towards a higher risk of bleeding complications. Specifically, compared with group O, the other three blood groups (A, B, and AB) demonstrated a modest, nonsignificant reduction in the likelihood of severe bleeding. This may be attributed to lower levels of von Willebrand factor and factor VIII in group O, both of which are critical for clot formation. 37 38

This research documented 37 distinct mutations associated with CFD, including 10 novel variants, and predicted their biological implications. However, the study has certain limitations. Long-term follow-up of patient families was not conducted, which is critical as asymptomatic individuals may develop bleeding or thrombotic symptoms years after diagnosis. Additionally, structural variants were not assessed due to limitations of the custom-designed NGS panel and Illumina platform, which are optimized for detecting single nucleotide variants and small insertions/deletions (indels) but lack the capability to capture larger genomic alterations. Although large deletions are rare in CFD, with only three major deletions (1238 bp, 11 kb, and 15 kb) reported to date. 39 40 41 Structural variants such as large deletions or duplications could contribute to the genetic heterogeneity of CFD and may play a role in misdiagnosis. Future studies should incorporate techniques such as multiplex ligation-dependent probe amplification, long-read sequencing, or cytogenetic methods to comprehensively identify structural variants, thereby enabling a more complete understanding of the genetics underlying CFD. 42 Despite this limitation, our findings significantly enhance the current knowledge of point mutations and small indels in CFD.

Overall, this single-center study provides an overview of the clinical, laboratory, and genetic features of CFD patients in China. The research identified a negative correlation between Fg∶C levels and bleeding severity. Additionally, due to specific physiological characteristics, female patients with CFD are at a higher risk of gynecological and pregnancy-related complications during their reproductive years. We further identified R35 of FGA and R301 of FGG as hotspot mutations among Chinese CFD patients. Furthermore, we confirmed that FGB:c.C130T: p.R44C is a mutation associated with thrombophilia.

## Notes

**What is known about this topic?**

- Congenital fibrinogen disorders (CFD) are characterized by bleeding episodes, with laboratory tests often revealing prolonged thrombin time (TT) while activated partial thromboplastin time (aPTT) and prothrombin time (PT) remain normal or slightly prolonged.
- The majority of genetic mutations associated with CFD are missense mutations, particularly concentrated in the FGA gene, followed by the FGB and FGG genes.

**What does this paper add?**

- This study represents the largest retrospective analysis of CFD patients in Asia, providing detailed insights into the genetic landscape and types of mutations associated with the disorder.
- It identifies a significant negative correlation between fibrinogen functional activity (FIB∶C) levels and bleeding severity, reinforcing the importance of functional tests over antigen levels in assessing bleeding risk.
- The research documents 37 distinct mutations related to CFD, including 10 new variants, and further suggests an association between the FGB:c.C130T: p.R44C mutation and thrombophilia, thereby enhancing the understanding of genetic factors influencing both bleeding and thrombotic risks in CFD patients.
