# Supplementary material for: Clinical and Genetic Characterization of 51 Patients with Congenital Fibrinogen Disorders from China
Source: Thromb Haemost. 2025 Jan 31;125(10):972–84. doi: 10.1055/a-2514-7520 (PMC12457006; doi:10.1055/a-2514-7520)
Supplement: Supplementary file 1 — Supplementary Material [file 10-1055-a-2514-7520-s24100518.pdf]

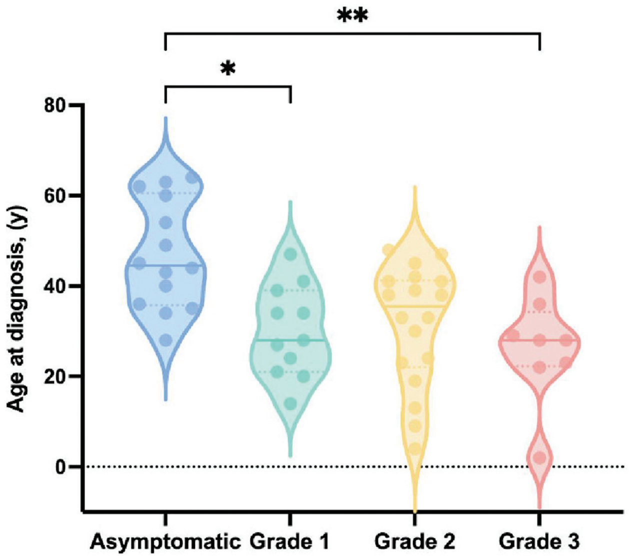

Supplementary Fig. S1 Age at diagnosis in different grades of severity. The solid line indicates the median, whereas the dashed line represents the interquartile range. The Kruskal–Wallis test was conducted to assess the differences in age at diagnosis across various severity grades. $^{*}p < 0.05$, $^{**}p < 0.01$.

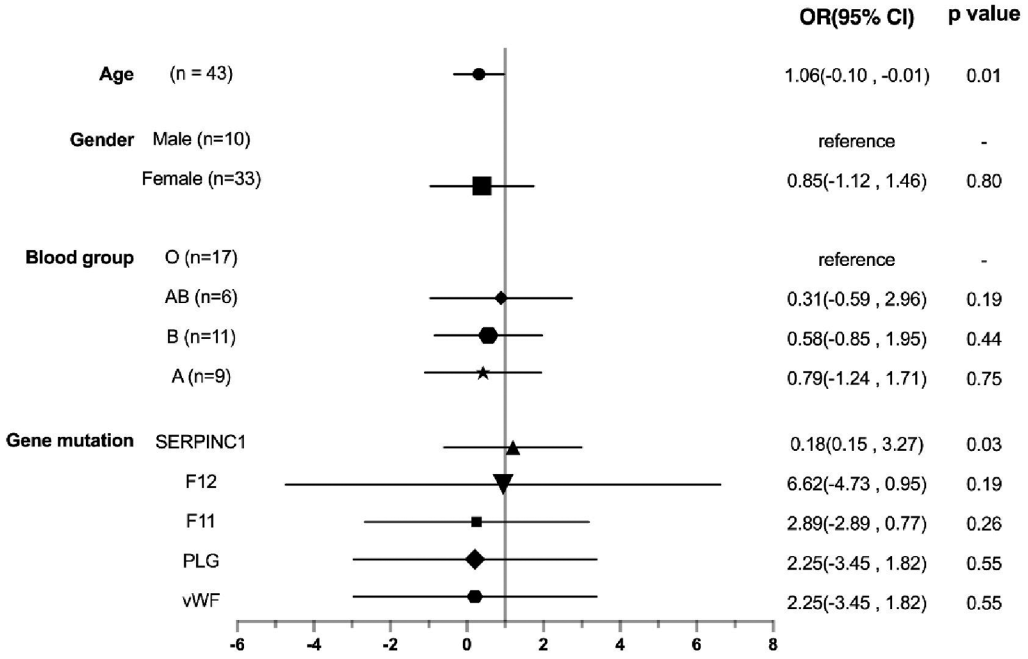

Supplementary Fig. S2 Forest plot of the impact on bleeding severity. The forest plot displays the odds ratios (ORs) and 95% confidence intervals (CIs) for the association of coagulation-related gene mutations (SERPINC1, F12, F11, PLG, vWF) and ABO blood type with bleeding severity. Statistical significance was considered at a p-value < 0.05.

 © 2025. The Author(s).
